# The impact of macrophages on endothelial cells is potentiated by cycling hypoxia: Enhanced tumor inflammation and metastasis

**DOI:** 10.3389/fonc.2022.961753

**Published:** 2022-09-28

**Authors:** Victor Delprat, Camille Huart, Olivier Feron, Fabrice Soncin, Carine Michiels

**Affiliations:** ^1^ Biochemistry and Cellular Biology Research Unit (URBC), Namur Research Institute for LIfe Sciences (NARILIS), University of Namur (UNamur), Namur, Belgium; ^2^ Pole of Pharmacology and Therapeutics (FATH 5349), Institut de recherche expérimentale et clinique, UCLouvain, Brussels, Belgium; ^3^ Laboratory for Integrated Micro Mechatronics Systems/Centre National de la Recherche scientifique- International Collaborative Research Center (LIMMS/CNRS-IIS) (Unité Mixte Internationale (UMI) 2820), Institute of Industrial Science, The University of Tokyo, Tokyo, Japan; ^4^ Centre National de la Recherche Scientifique/International Collaborative Research Center (CNRS/IIS/COL) Lille University Seeding Microsystems in Medecine in Lille (SMMiL) – European-Japanese Technologies against Cancer-E Project, CNRS Délégation Nord-Pas de Calais et Picardie, Cedex, France; ^5^ Institut pour la Recherche sur le Cancer de Lille (IRCL), Université de Lille, CNRS, Lille, France

**Keywords:** cycling hypoxia, macrophages, endothelial cells, cancer cells, cancer

## Abstract

Cycling hypoxia (cyH), neo-angiogenesis, and tumor-associated macrophages are key features of the tumor microenvironment. In this study, we demonstrate that cyH potentiates the induction by unpolarized and M1-like macrophages of endothelial inflammatory phenotype and adhesiveness for monocytes and cancer cells. This process triggers a positive feedback loop sustaining tumor inflammation. This work opens the door for innovative therapeutic strategies to treat tumor inflammation and metastasis.

In cancers, the interaction between macrophages and endothelial cells (ECs) regulates tumor inflammation and metastasis. These cells are both affected by cycling hypoxia (cyH), also called intermittent hypoxia, a feature of the tumor microenvironment. cyH is also known to favor tumor inflammation and metastasis. Nonetheless, the potential impact of cyH on the dialog between macrophages and ECs is still unknown. In this work, the effects of unpolarized, M1-like, and M2-like macrophages exposed to normoxia, chronic hypoxia (chH), and cyH on endothelial adhesion molecule expression, pro-inflammatory gene expression, and EC adhesiveness for monocytes and cancer cells were investigated. cyH increased the ability of unpolarized and M1-like macrophages to induce EC inflammation and to increase the expression of the EC endothelial adhesion molecule ICAM1, respectively. Unpolarized, M1-like, and M2-like macrophages were all able to promote EC adhesive properties toward cancer cells. Furthermore, the ability of macrophages (mostly M1-like) to shift EC phenotype toward one allowing cancer cell and monocyte adhesion onto ECs was potentiated by cyH. These effects were specific to cyH because they were not observed with chH. Together, these results show that cyH amplifies the effects of macrophages on ECs, which may promote tumor inflammation and metastasis.

## 1 Introduction

The tumor microenvironment (TME) is composed of cancer cells and stromal cells (e.g., fibroblasts, and endothelial and immune cells), which are exposed to several physicochemical stresses. These stromal cells are strongly involved in the modulation of tumor growth, metastasis, and tumor inflammation (1–3). One of the most important physicochemical features, which modifies the TME, is the reduction of oxygenation, a phenomenon called hypoxia. In tumors, two types of hypoxia occur: cycling hypoxia (cyH) and chronic hypoxia (chH) (4). chH is the result of uncontrolled proliferation of cancer cells, which leads to some cells being too far from blood vessels to be oxygenated. CyH, also called acute, intermittent, or cyclic hypoxia, is due to intermittent erythrocyte flow into the tumor blood vessels, which leads to periods of hypoxia followed by periods of reoxygenation. Another cause of cyH in cancer is sleep apnea, in which periods of hypoxia/reoxygenation are shorter and more numerous. cyH (and chH) strongly regulates Hypoxia-inducible factor-1alpha (HIFα) stability and activity (5, 6). cyH is involved in the promotion of several hallmarks of tumor, such as resistance to therapy, metastasis, angiogenesis, cancer development, genomic instability, and, which we previously described, tumor inflammation (5, 7–9). Some of these features are promoted to a higher extent by cyH than chH (4, 5). Previously in the laboratory, we showed that cyH induces endothelial cell (EC) activation *via* nuclear factor-kappa B (NF-kB) pathway activation, hence promoting tumor inflammation (8). Tumor chronic inflammation is a hallmark of cancer and is involved in tumor development and progression (10). Tumor inflammation is strongly regulated by immune cells, and tumor inflammation regulates the phenotype of a majority (if not all) of cell types of the TME. Accordingly, we showed that cyH favors inflammatory phenotype in unpolarized and M1-like macrophages, which could be one explanation of cyH-induced tumor inflammation (9). Furthermore, it is well known that cyH promotes metastasis (4, 11). Several reports showed that the exposure of mice carrying spontaneous or induced tumors to cyH increased the metastatic spread of cancer cells (11). Both metastases and tumor inflammation are hallmarks of cancer (12, 13). Metastasis is involved in most of the cancer-related deaths, and hence, it is very important to understand this process. Inflammation not only is a cause of genetical instability but also enhances angiogenesis, cancer cell proliferation, and survival as well as metastasis (13, 14). Hence, metastasis and inflammation are linked to poor disease outcome.

Stromal cells are involved in the promotion of metastasis and inflammation. Among these cells, tumor-associated macrophages (TAMs) and ECs are strongly involved in these processes (15–19). In tumors, macrophages originate mostly from blood monocytes that infiltrate the tumors and then differentiate in macrophages. Macrophages are usually classified according to the M1 and M2 polarization axis. M1 macrophages are pro-inflammatory and induced by Interferon gamme IFNγ; lipopolysaccharide (LPS) stimulation, whereas M2 macrophages are anti-inflammatory and induced notably by interleukin-4 (IL-4) and/or IL-13 stimulation (20). The endothelium is composed of an EC monolayer that lines the inner side of blood vessels in direct contact with the circulation. ECs play essential roles in the exchanges of gas and metabolites between blood and tissues, in the regulation of vascular dilation, in thrombosis, in angiogenesis, and in the immune response (17, 21). Blood vessels are essential to the growth of most solid tumors because they sustain a constant supply of nutriments and oxygen to the tumors (22). Furthermore, ECs are strongly involved in tumor metastasis. Indeed, the metastasis process corresponds to the spread of cancer cells from a primary tumor site into a secondary site. This process is composed of several steps, and ECs participate in some of them such as cancer cell migration toward blood vessels, intravasation, and extravasation (18, 23, 24). In tumors, ECs and macrophages strongly interact with each other in a bi-directional dialog that influences the tumor progression (19). Indeed, TAMs favor angiogenesis and lymphangiogenesis and shift EC phenotype toward one promoting cancer cell intravasation and extravasation (19, 25, 26). On the other hand, ECs are critically involved in the regulation of monocyte/macrophage infiltration in the tumor, and infiltrated monocytes/macrophages enhance cancer cell extravasation and seeding as well as regulate tumor inflammation (16, 17, 19). At the molecular level, the transmigration of immune cells through the endothelium is well described and depends on a complex set of interactions between leukocyte and EC membrane receptors (27, 28). In case of endothelial activation, ECs strongly express leukocyte adhesion receptors at their luminal face, such as E-selectin, intercellular adhesion molecule 1 (ICAM1), and vascular cell adhesion molecule 1 (VCAM1). These receptors favor the rolling, arrest, firm adhesion, and extravasation of immune cells from the circulation toward the tissues (27). On the other hand, cancer cell binding onto endothelium is less well described. To disseminate, the intravasation and the extravasation (in blood or lymphatic system) of cancer cells are needed. Intravasation and extravasation are two different processes, because cancer cells cross ECs from opposite side, and that ECs have an apico-basal polarity. ICAM1, E-selectin, VCAM1, neuronal cadherin (N-cadherin), and integrins are among the best characterized molecules of the ECs involved in cancer cell adhesion onto endothelium and cancer cell extravasation (18, 24, 29–31). Cancer cells intravasation is less well understood. Several cells of the TME regulate cancer cell intravasation, such as neutrophils and TAMs.

The effect of cyH on the effects of macrophages on ECs is still unknown. Because cyH, macrophages, and ECs are involved in tumor metastasis and inflammation, it is very important to investigate the effect of cyH on the interaction between macrophages and ECs. In this work, the impact of unpolarized, M1-like, and M2-like macrophages exposed to cyH on EA.hy926 cell (EC lineage) and on primary human umbilical vein EC (HUVEC) phenotype was studied. Results showed that the adhesion of monocytes onto ECs incubated with M1-like cyH medium was strongly enhanced compared with that of ECs incubated with M1-like N and M1-like chH media. Furthermore, macrophages exposed to cyH (i) increased pro-inflammatory cytokine and protein expression in EA.hy926 cells and (ii) increased the mRNA expression and the protein abundance of ICAM1 in ECs compared with macrophages exposed to N or chH. The adhesion of breast cancer cells onto ECs was enhanced by unpolarized, M1-like, and M2-like macrophages, and the effect of M1-like macrophages was potentiated by cyH. Together, these results show that cyH initiates an amplification positive feedback loop between TAMs and ECs to maintain tumor inflammation and metastasis.

## 2 Materials and methods

### 2.1 Cell culture

THP-1 monocytes (American Type Culture Collection (ATCC); TIB-202; RRID : CVCL_0006) were cultured in RPMI 1640 medium (21875, Gibco), supplemented with 10% heat-inactivated fetal bovine serum (HIS), 10 mM HEPES, 1 mM pyruvate, glucose (2.5 g/L), and 50 μM β-mercaptoethanol. EA.hy926 cells (RRID : CVCL_3901) and primary HUVECs were from ATCC (CRL-2922) and from Lonza (CC-2519), respectively. EA.hy926 ECs were cultivated in DHGL-1 medium (high-glucose Dulbecco's Modified Eagle's Medium (DMEM), low NaHCO_3_, without sodium pyruvate) supplemented with 10% fetal bovine serum. HUVECs were grown in EGM-2 medium (Lonza), until passage 7. MDA-MB-231 breast cancer cells (ATCC; HTB-26; RRID : CVCL_0062) were cultivated in high-glucose DMEM (Gibco), 10% HIS, and 0.5 mM glutamine.

All cell lines have been authenticated using short tandem repeat profiling within the last 3 years. All experiments were performed with mycoplasma-free cells.

### 2.2 Differentiation and polarization of THP-1 monocytes into unpolarized, M1-like, and M2-like macrophages and macrophage-derived conditioned medium formation

The protocol used to differentiate and polarize human THP-1 monocytes into unpolarized, M1-like, and M2-like macrophages was set up in (20) and used as in (9). Briefly, THP-1 monocytes were incubated with 150 nM Phorbol 12-myristate 13-acetate (PMA) for 24 h and were then left untreated for 24-h period in complete RPMI medium. This allowed for the differentiation of monocytes into unpolarized macrophages characterized by a decrease in monocyte marker expression and an increase in macrophage marker expression (20). The unpolarized macrophages were left untreated or were polarized into M1-like macrophages *via* 24-h incubation with LPS (10 pg/ml; Sigma; L8630) and IFNγ (20 ng/ml; R&D Systems) or were polarized into M2-like macrophages *via* 48-h incubation with IL-4 (20 ng/ml; R&D Systems) and IL-13 (20 ng/ml; R&D Systems). The proportion of M1 macrophages in the cell population after polarization with LPS and IFNγ has been quantified by fluorescence activated cell sorting (FACS), using human leukocyte antigen (HLA) as the marker for M1 phenotype. There were 83.1% of cells positive for HLA in the M1-like population compared with 17% in unpolarized macrophages and to 5.9% in the M2-like population (data not shown).

Unpolarized, M1-like, and M2-like macrophages were then exposed to normoxia, chH, and cyH for 6 h and were then left for 16 h in normoxic air (21% O_2_). chH incubation corresponded to 6 h of 1% O_2_, whereas cyH corresponded to four cycles of 1% O_2_ for 1 h and 21% O_2_ for 30 min. The incubation of cells in hypoxia was performed as in (9). In this study, normoxia and the cyH reoxygenation were performed by exposing cells to atmospheric air (21% O_2_) and not physioxia. Nonetheless, the hypoxia value that we used was physiologically relevant because O_2_ saturation in tumor is comprised between 1% and 2% (8–15 mmHg) O_2_ in a majority of solid tumors (32).

During this 22-h (6 h + 16 h) incubation period, macrophages were incubated in CO_2_-independent medium (18015, Gibco) supplemented with 4 mM glutamine (G8540, Sigma) and glucose (3.75 g/L; 6877, Roth). After incubation, conditioned media were harvested, and the pH of each medium was adjusted to that of CO_2_-independent medium pH ± 0.02. As shown previously, the polarization state of macrophages incubated during this period of time (6 h + 16 h) in N, chH, or cyH was not modified. Indeed, unpolarized, M1-like, and M2-like macrophages remained, respectively, unpolarized, M1-like, and M2-like macrophages after this incubation period (9). Conditioned media were either stored at 4°C for a maximum period of 3 days and used for experiments or were frozen in liquid nitrogen (snap-freeze) and stored at −70°C and were used afterward for experiments. The media that were stored at 4°C no more than 3 days or that were snap-frozen and stored at −70°C showed the same biological effects (data not shown).

### 2.3 Incubation of endothelial cells with macrophage-conditioned media

EA.hy926 cells were incubated with 100% of macrophage-conditioned media, during the corresponding incubation time. Before seeding HUVECs, plates were coated with fibronectin (1 µg/ml; 1030-FN, R&D Systems) for 45 min at 37°C. Fibronectin was removed, and plates were rinsed with phosphate buffered saline (PBS). Then, HUVECs were seeded and incubated for 24 h at 37°C and 5% CO_2_ prior to incubation with 23.5% EGM-2, 1.5% HIS, and 75% macrophage-conditioned media, during the corresponding incubation time.

### 2.4 Immunofluorescence labeling

EA.hy926 cells and HUVECs were seeded in 24-well plates at 7,500 and 25,000 cells per well, respectively. ECs were incubated for 24 h at 37°C and 5% CO_2_ prior to incubation for 48 h in 1 ml of control medium or macrophage-conditioned media as indicated in Section 2.3. As a positive control, cells were incubated for 16 h with tumor necrosis factor-alpha (TNFα) (1 ng/ml; 210-TA, R&D Systems). After incubation, ECs were fixed for 15 min in 400 µl of 4% paraformaldehyde in PBS. Cells were washed three times in PBS and were blocked in PBS containing 2% of bovine serum albumin (BSA) for 30 min. Then, cells were incubated overnight (O/N) at 4°C with anti-ICAM1 primary antibody (diluted 1:60 in 2% BSA in PBS; #BBA3, R&D Systems). Cells were washed for 30 min in 2% BSA in PBS and were incubated with the secondary antibody (Alexa Fluor 488–conjugated anti-rabbit immunoglobulin G (IgG) antibody; diluted 1:1,000 in 2% BSA in PBS; #A1103, Molecular Probes). Cells were then incubated with 4',6-diamidino-2-phenylindole (DAPI) (diluted 1:2,500 in PBS; 10236276001, Sigma) to stain the nucleus. The coverslips were mounted in Mowiol (Sigma), and pictures were taken using a confocal microscope (SP5, Leica). Pictures were analyzed using ImageJ software.

### 2.5 MTT assay

EA.hy926 cells and HUVECs were seeded in 24-well plates at 50,000 and 25,000 cells per well, respectively. ECs were then incubated for 24 h at 37°C and 5% CO_2_ prior to incubation for 48 h in 500 µl of control medium or macrophage-conditioned media, as indicated in Section 2.3. Then, 500 µl of MTT solution (2.5 mg/ml; M2128, Sigma) in PBS was added, and cells were incubated for 2 h at 37°C and 5% CO_2_. Media were removed, and cells were lysed in 1 ml of dimethyl sulfoxide (DMSO) (A994, Roth) and under slight agitation, for 1 h. Afterward, absorbance was read at 570 nm.

### 2.6 Enzyme-linked immunosorbent assay (ELISA)

EA.hy926 cells and HUVECs were seeded in 24-well plates at 50,000 and 25,000 cells per well, respectively. ECs were then incubated for 24 h at 37°C and 5% CO_2_ prior to incubation in 1 ml of control medium or macrophage-conditioned media, as indicated in Section 2.3. In parallel, 1 ml of each corresponding conditioned medium was incubated at 37°C during the same period (24 h). Supernatants were harvested and centrifuged at 200*g* for 5 min at 4°C. Supernatants were stored at −70°C and used afterward for cytokine quantification. In parallel, ECs were lysed with 200 µl of NaOH 0.5 N and frozen at −20°C. The concentration of IL-6 and IL-8 in supernatants was analyzed using human quantikine ELISA kits (R&D Systems), which were used according to the manufacturer’s indications. The concentration of endothelial cell–specific molecule 1 (ESM1) protein in supernatant was analyzed using a human ESM1 ELISA kit (ab213776, Abcam) and used according to the manufacturer’s indications. To quantify only the protein secreted by ECs, the proteins contained in the macrophage supernatant were subtracted from each corresponding EC supernatant. Cytokine concentrations (picograms per milliliter) were normalized by total protein concentration (micrograms) contained in ECs and macrophages determined by the Folin method after 200 µl (ECs) or 1 ml (macrophages) of NaOH 0.5 N lysis.

### 2.7 Western bot

EA.hy926 cells were seeded in 25-cm (2) flasks at 5,000 cells/cm (2). EA.hy926 cells were then incubated for 24 h at 37°C and 5% CO_2_ prior to incubation in 5 ml of control media or macrophage-conditioned media. Then, cells were lysed, and Western blots for ICAM1 were performed as in (8). Antibodies used are listed in Supplementary **Table S1**.

### 2.8 Reverse transcriptase-quantitative polymerase chain reaction (RT-qPCR)

EA.hy926 cells and HUVECs were respectively seeded in six-well plates and 12-well plates at 250,000 and 30,000 cells per well, respectively. ECs were then incubated for 24 h at 37°C and 5% CO_2_ prior to incubation for 24 h in 2 or 1 ml of control medium or macrophage-conditioned media, as indicated in Section 2.3. Total RNA extraction was performed using the ReliaPrep RNA Tissue Miniprep Systems (Z6111, Promega) according to the manufacturer’s instruction. Total RNA extract was quantified using nanophotometer (N60, IMPLEN), and 2 µg of RNA was reverse-transcribed using the GoScript Reverse Transcription Mix, Oligo(dT) (#A2791, Promega). qPCR was performed as in (9). RPL13A and β-2-microglobulin genes were used for normalization in EA.hy926 cells and HUVEC samples, respectively.

### 2.9 THP-1 monocyte adhesion onto endothelial cells

EA.hy926 cells or HUVECs were grown to confluence in 96-well plates and 24-well plates, respectively. ECs were incubated with 1 ml or 200 µl of control medium or macrophage-conditioned media, as indicated in Section 2.3, respectively. THP-1 monocytes were rinsed in RPMI without phenol red (11835, Gibco) and were labeled with 5 µM calcein-AM at a concentration of 5.10 (6) cells/ml, for 30 min at 37°C and 5% CO_2_. EA.hy926 cells and HUVECs were rinsed with RPMI and with EGM-2, respectively. We verified that there was a clear linearity between the measured fluorescence and the number of labeled cells added to the wells (data not shown). TNFα at 1 ng/ml for 6 h was used as positive control. THP-1 monocytes were then seeded and incubated for 1 h with EA.hy926 cells or HUVECs, at a density of 50,000 cells per well for EA.hy926 cells or of 100,000 cells per well for HUVECs. Directly after THP-1 seeding, plates were read using fluorimeter (485-nm excitation and 520-nm emission) to ensure equal seeding between each well (INPUT). After 1 h, cells were washed with RPMI and then read using fluorimeter (485-nm excitation and 520-nm emission). Each well was normalized according to INPUT, and data were presented as % of adherent THP-1 monocytes.

### 2.10 MDA-MB-231 breast cancer cell adhesion onto HUVECs

HUVECs were grown to confluence in 24-well plates. HUVECs were incubated with 1 ml of control medium or macrophage-conditioned media, as indicated in Section 2.3. MDA-MB-231 cells were rinsed in RPMI without phenol red and were labeled with 5 µM calcein-AM (V13181, ThermoFisher) at a concentration of 5.10 (6) cells/ml, for 30 min at 37°C. We verified that there was a clear linearity between the measured fluorescence and the number of labeled cells added to the wells (data not shown). HUVECs were rinsed with EGM-2, and MDA-MB-231 cells were then seeded and incubated for 1 h on HUVECs, at a density of 40,000 cells per well. Directly after MDA-MB-231 cell seeding, plates were read using fluorimeter (485-nm excitation and 520-nm emission) to ensure equal seeding between each well (INPUT). To make sure that the monocytes were indeed adhering to the EC monolayer and not to fibronectin in between retracted cells, all the plates were manually checked using phase contrast microscopy. We never observed that the EC monolayers were disrupted in all these experiments. After 1 h, cells were washed with RPMI and then read using fluorimeter (485-nm excitation and 520-nm emission). Each well was normalized according to INPUT, and data were presented as % of adherent MDA-MB-231 cells.

### 2.11 Expression data retrieval and analysis

CD68, CD80, CD86, CD163, CD206, ICAM1, and VCAM1 expression was extracted from https://xena.ucsc.edu/. A total of 1,092 breast cancer samples were analyzed. Data analyses were processed using R studio. The Pearson correlation coefficients (r-values) and p-values were calculated for each combination.

### 2.12 Statistical analysis

Data are reported as mean ± 1 SEM. Statistical analyses were performed using SigmaPlot software. Corresponding statistical tests are outlined in the figure legends.

## 3 Results

In this work, we investigated the impact of THP-1–derived unpolarized, M1-like, and M2-like macrophages exposed to cyH on EA.hy926 cells and HUVECs, respectively. The experiments were performed initially on the EA.hy926 cell line and confirmed on primary HUVECs. To this purpose, THP-1 monocytes were differentiated and polarized in unpolarized, M1-like, and M2-like macrophages as in (9). To produce macrophage-conditioned media, unpolarized, M1-like, and M2-like macrophages were exposed to N, chH, and cyH for 6 h and were left for 16 h in N. As shown previously, unpolarized, M1-like, and M2-like macrophages remained, respectively, unpolarized, M1-like, and M2-like macrophages after this incubation period (9). Macrophage-conditioned media were harvested, and ECs were incubated in these media during the indicated times. Effects of macrophages exposed to cyH on ECs were systematically compared with the effects of macrophages exposed to chH.

### 3.1 Cycling hypoxia amplifies the effect of macrophages on EC adhesiveness for monocytes

First, we tested whether macrophage media were toxic to ECs by assessing cell viability using a MTT assay. No effects of conditioned media on cell viability were observed when incubating EA.hy926 cells or HUVECs with macrophage media for 48 h (**Supplementary Figure 1**).

**Figure 1:**
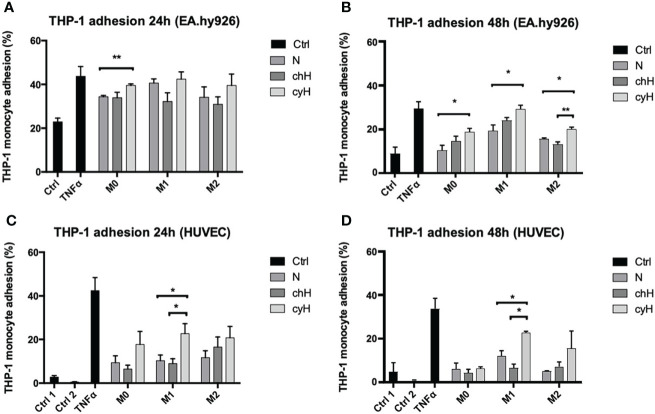
Adhesion of THP-1 monocytes on EA.hy926 cells or HUVECs incubated with macrophage-conditioned media. THP-1–derived unpolarized, M1-like, and M2-like macrophages were exposed to normoxia (N), chronic hypoxia (chH), or cycling hypoxia (cyH) for 6 h and were then left for 16 h in normoxic air to produce macrophage-conditioned media. Confluent monolayer of EA.hy926 cells **(A, B)** and HUVECs **(C, D)** were incubated for 24 h **(A, C)** or 48 h **(B, D)** with macrophage-conditioned media. Thereafter, the adhesion of THP-1 monocytes **(A–D)** on endothelial cells was assessed by adhesion assay (n = 3, mean ± 1 SEM). Ctrl 1 and Ctrl 2 correspond to endothelial cells incubated with CO_2_-independent medium or EGM-2 medium, respectively. Incubation of endothelial cells with TNFα (1 ng/ml) for 6 h was used as positive control. Statistical analysis was performed using Student’s t test. *p < 0.05; **p < 0.01.

In a previous study, we showed that cyH amplified the pro-inflammatory phenotype of unpolarized and M1-like macrophages (9). Therefore, in this study, we tested the functional impact of the incubation of EA.hy926 cells and HUVECs with cyH-exposed macrophage media on undifferentiated THP-1 monocyte adhesion onto the endothelium (**Figure 1** and **Supplementary Figure 2**). To this purpose, EA.hy926 cells and HUVECs were incubated for 24 or 48 h with the media of unpolarized, M1-like, and M2-like macrophages exposed to N, chH, and cyH. Afterward, the adhesion of THP-1 monocytes onto EC monolayer was assessed. The adhesion of THP-1 monocytes onto EA.hy926 cells was slightly and significantly increased when EA.hy926 cells were incubated for 24 h with the unpolarized cyH medium compared with unpolarized N medium (**Figure 1A**). After 48-h incubation, THP-1 monocyte adhesion onto EA.hy926 was increased when treated with unpolarized cyH, M1-like cyH, and M2-like cyH media compared with the respective normoxic media (**Figure 1B**). The adhesion of THP-1 monocytes onto HUVECs was significantly increased in HUVECs incubated for 24 and 48 h with M1-like cyH medium compared with HUVECs incubated for 24 and 48 h with M1-like N and M1-like chH media (**Figures 1C, D** and **Supplementary Figure 2**). Interestingly, the adhesion of monocytes onto both EC types was more increased when cells were exposed for 48 h to M1-like macrophage media, compared with unpolarized and M2-like macrophage media. Together, these results showed that cyH amplifies the effect of macrophages on EC adhesiveness for monocytes.

### 3.2 Cycling hypoxia amplifies the effect of unpolarized macrophages on EC inflammatory protein expression and secretion

We then compared the effects of cyH-exposed macrophage media with N- and chH-exposed macrophage media on EA.hy926 cells and HUVEC mRNA expression and secretion of pro-inflammatory cytokines (IL-6 and IL-8) and proteins (ESM1). ESM1 (EC-specific molecule-1), also known as endocan, is a soluble dermatan sulfate proteoglycan. It is secreted by various cell lines and, more particularly, by human vascular ECs. Its secretion is increased upon stimulation by cytokines and pro-angiogenic factors. It is recognized as a biomarker of endothelial dysfunction and inflammation (33, 34).

**Figure 2:**
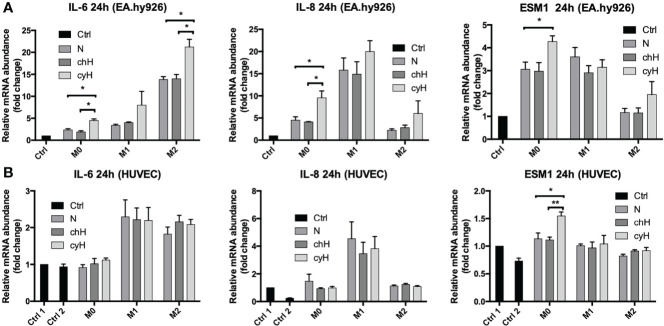
Endothelial pro-inflammatory mRNA expression in EA.hy926 cells and HUVECs incubated for 24 h with macrophage-conditioned media. THP-1–derived unpolarized, M1-like, and M2-like macrophages were exposed to normoxia (N), chronic hypoxia (chH), or cycling hypoxia (cyH) for 6 h and were then left for 16 h in normoxic air to produce macrophage-conditioned media. Thereafter, EA.hy926 cells **(A)** and HUVECs **(B)** were incubated for 24 h with macrophage-conditioned media and their mRNA expression for IL-6, IL-8, and ESM1 was assessed by RT-qPCR (n = 4, mean ± 1 SEM). Ctrl 1 and Ctrl 2 correspond to endothelial cells incubated with CO_2_-independent medium or EGM-2 medium, respectively. Statistical analysis was performed using Student’s t test. *p < 0.05; **p < 0.01.

**Figure 3:**
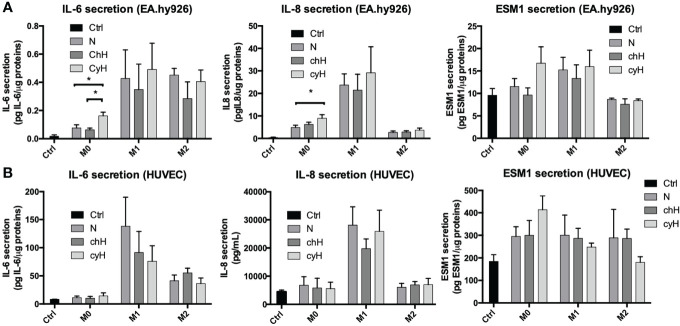
Endothelial pro-inflammatory protein secretion in EA.hy926 cells and HUVECs incubated for 24 h with macrophage-conditioned media. THP-1–derived unpolarized, M1-like, and M2-like macrophages were exposed to normoxia (N), chronic hypoxia (chH), or cycling hypoxia (cyH) for 6 h and were then left for 16 h in normoxic air to produce macrophage-conditioned media. Thereafter, EA.hy926 cells **(A)** and HUVECs **(B)** were incubated for 24 h with macrophage-conditioned media and the secretion of IL-6, IL-8, and ESM1 in the endothelial cells was assessed by ELISA (n = 3, mean ± 1 SEM). Ctrl corresponds to endothelial cells incubated with CO_2_-independent medium. Statistical analysis was performed using Student’s t test. *p < 0.05.

To this purpose, EA.hy926 cells and HUVECs were incubated for 24 h with macrophage media, and the mRNA expression and secretion of the pro-inflammatory cytokines in ECs were assessed by qPCR and ELISA, respectively. To quantify only the proteins secreted by ECs, the protein concentration contained in the macrophage supernatant were subtracted from the one corresponding to each EC supernatant. The mRNA expression of IL-6, IL-8, and ESM1 was significantly higher in EA.hy926 cells incubated with unpolarized cyH medium than in EA.hy926 cells incubated with unpolarized N or unpolarized chH media (**Figure 2A**). These results were consistent with protein profiles because the secretion of IL-6 and IL-8 was significantly higher in EA.hy926 cells incubated with unpolarized cyH medium than in EA.hy926 cells incubated with unpolarized N and unpolarized chH media (**Figure 3A**). Furthermore, ESM1 protein secretion tends to increase in EA.hy926 cells incubated with unpolarized cyH medium compared with unpolarized N and unpolarized chH media, although these differences did not reach statistical significance (**Figure 3A**). EA.hy926 cells incubated with M2-like cyH medium displayed a significantly higher IL-6 expression compared with EA.hy926 cells incubated with M2-like N and M2-like chH media. In HUVECs, only ESM1 mRNA expression was significantly increased by unpolarized cyH medium compared with the unpolarized N and unpolarized chH media (**Figure 2B**). Consistently, ESM1 protein secretion tends to increase in HUVECs incubated with unpolarized cyH medium compared with HUVECs incubated with unpolarized N and unpolarized chH media, but this effect did not reach statistical significance (**Figure 3B**). In addition to pro-inflammatory cytokines, the expression of anti-inflammatory cytokines was also assessed. EA.hy926 cells did not express IL-10 whatever the conditions. These cells did express transforming growth factor-beta (TGF-β), but TGF-β expression was not affected by macrophage-conditioned media (data not shown).

The impact of macrophages, on their own, on the expression and secretion of cytokines by ECs was different according to the macrophage phenotype. Indeed, the expression of IL-8 was strongly increased by M1 macrophage media. Accordingly, the expression and secretion of IL-8 by HUVECs were only modulated by M1-like macrophage media in HUVECs. The expression of IL-6 by EA.hy926 cells was strongly increased by M2-like macrophages, whereas this was upregulated by M1-like and M2-like macrophages in HUVECs. The secretion of IL-6 was increased by M1-like and M2-like macrophages in both EC types. In conclusion, unpolarized exposed to cyH induced a pro-inflammatory phenotype in EA.hy926 cells and HUVECs, and this pro-inflammatory phenotype was stronger in EA.hy926 cells than in HUVECs.

### 3.3 Cycling hypoxia amplifies the effect of macrophages on the induction of endothelial ICAM1 expression

**Figure 4:**
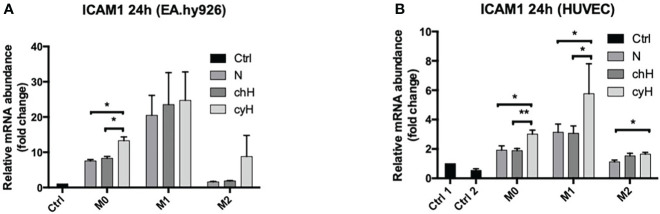
ICAM1 mRNA expression in EA.hy926 cells and HUVECs incubated with macrophage-conditioned media. THP-1–derived unpolarized, M1-like, and M2-like macrophages were exposed to normoxia (N), chronic hypoxia (chH), or cycling hypoxia (cyH) for 6 h and were then left for 16 h in normoxic air to produce macrophage-conditioned media. Thereafter, EA.hy926 cells **(A)** and HUVECs **(B)** were incubated for 24 h with macrophage-conditioned media. mRNA expression of ICAM1 in EA.hy926 cells **(A)** and HUVECs **(B)** was assessed by RT-qPCR (n = 4, mean ± 1 SEM). Ctrl 1 corresponds to endothelial cells incubated with CO_2_-independent medium. Ctrl 2 corresponds to endothelial cells incubated with EGM-2 medium. Statistical analysis was performed using Student’s t test. *p < 0.05; **p < 0.01.

Because the adhesion of THP-1 monocytes on ECs was increased by macrophage cyH media compared with macrophage N and chH media, we studied the impact of macrophages on the expression and protein abundance of EC adhesion molecules ICAM1, VCAM1, and E-selectin. To this purpose, EA.hy926 cells and HUVECs were incubated with unpolarized, M1-like, and M2-like macrophage media exposed to N, chH, and cyH. mRNA expression of endothelial adhesion molecules was assessed after 6-h (**Supplementary Figure 3B**) and 24-h (**Figure 4** and **Supplementary Figure 3A**) incubation. The mRNA expression levels of adhesion molecules were higher in HUVECs and EA.hy926 cells incubated with M1-like media compared with HUVECs or EA.hy926 incubated with M2-like media (**Figure 4** and **Supplementary Figures 3A, B**), except for VCAM1 expression in HUVECs incubated for 24 h with media, which was not different in HUVECs incubated with M1-like macrophage media compared with HUVECs incubated with M2-like macrophage media (**Supplementary Figure 3A**). This is consistent with a study in which endothelial adhesion molecule expression in ECs was shown to be more strongly induced by M1-like macrophages than M2-like macrophages (35). Interestingly, ICAM1 mRNA expression was significantly higher in EA.hy926 cells incubated for 24 h with unpolarized cyH medium, compared with EA.hy926 cells incubated for 24 h with unpolarized N or unpolarized chH media (**Figure 4A**). Furthermore, ICAM1 mRNA expression in HUVECs was significantly increased by unpolarized cyH, M1-like cyH, and M2-like cyH media compared with the respective normoxic macrophage media (**Figure 4B**). In HUVECs, the expression of VCAM1 or E-selectin was not modified by cyH-exposed macrophage media compared with the related N- or chH-exposed macrophage media (**Supplementary Figures 3A, B**). On the other hand, E-selectin expression was significantly higher in HUVECs incubated for 6 h with M2-like cyH medium compared to HUVECs incubated for 6 h with M2-like N or M2-like chH media (**Supplementary Figure 3B**).

**Figure 5:**
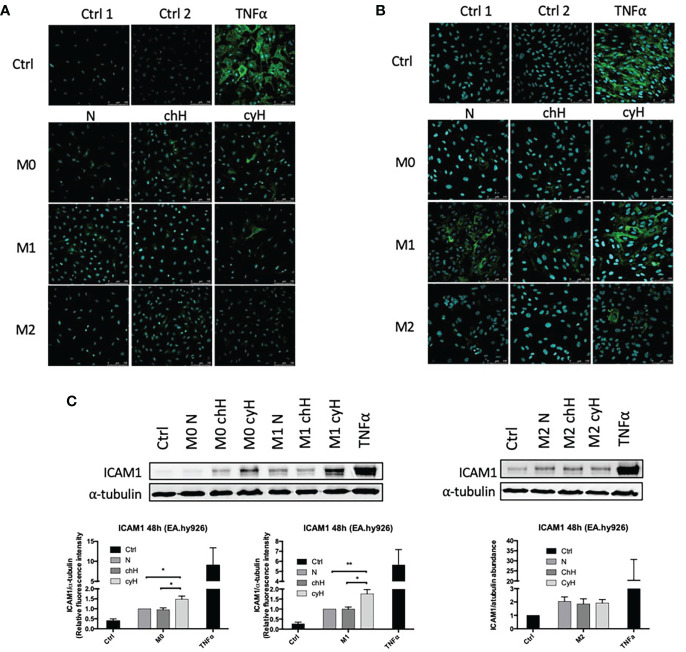
ICAM1 protein abundance in EA.hy926 cells and HUVECs incubated with macrophage-conditioned media. THP-1–derived unpolarized, M1-like, and M2-like macrophages were exposed to normoxia (N), chronic hypoxia (chH), or cycling hypoxia (cyH) for 6 h and were then left for 16 h in normoxic air to produce macrophage-conditioned media. Thereafter, EA.hy926 cells and HUVECs were incubated for 48 h with macrophage-conditioned media. ICAM1 protein abundance in EA.hy926 cells **(A)** and HUVECs **(B)** was analyzed by immunofluorescence (n = 2). ICAM1 protein abundance in EA.hy926 cells **(C)** was analyzed by western blot (n = 3, mean ± 1 SEM). Ctrl 1 corresponds to endothelial cells incubated with CO_2_-independent medium. Ctrl 2 corresponds to endothelial cells incubated with DHGL-1 **(A)** or EGM-2 **(B)**. Incubation of endothelial cells with TNFα (1 ng/ml) for 16 h was used as positive control. Statistical analysis was performed using Student’s t test. *p < 0.05; **p < 0.01.

Because ICAM1 mRNA expression in ECs was increased by cyH-exposed macrophage, we studied whether ICAM1 protein abundance was correspondingly increased (**Figure 5**). In EA.hy926 cells incubated for 48 h with macrophage media, ICAM1 protein abundance was significantly increased by unpolarized cyH and M1-like cyH media compared with the respective N-exposed macrophage media, whereas no differences were observed when ECs were incubated with M2-like macrophage media (**Figures 5A, C** and **Supplementary Figure 4A**). ICAM1 protein abundance was noticeably higher in EA.hy926 cells incubated for 24 h with M1-like media compared with EA.hy926 cells incubated with M2-like media (**Supplementary Figure 5**). No differences in ICAM1 protein abundance were observed in EA.hy926 cells incubated for 24 h with cyH-exposed macrophage media compared with N- or chH-exposed macrophage media (**Supplementary Figure 5**). In HUVECs, ICAM1 protein abundance was slightly increased by M1-like cyH medium and strongly increased by M2-like cyH medium compared with the respective N and chH media (**Figure 5B** and **Supplementary Figure 4B**).

### 3.4 Macrophages increase the adhesiveness of endothelial cells for MDA-MB-231 breast cancer cells, and this effect is potentiated by cyH

**Figure 6:**
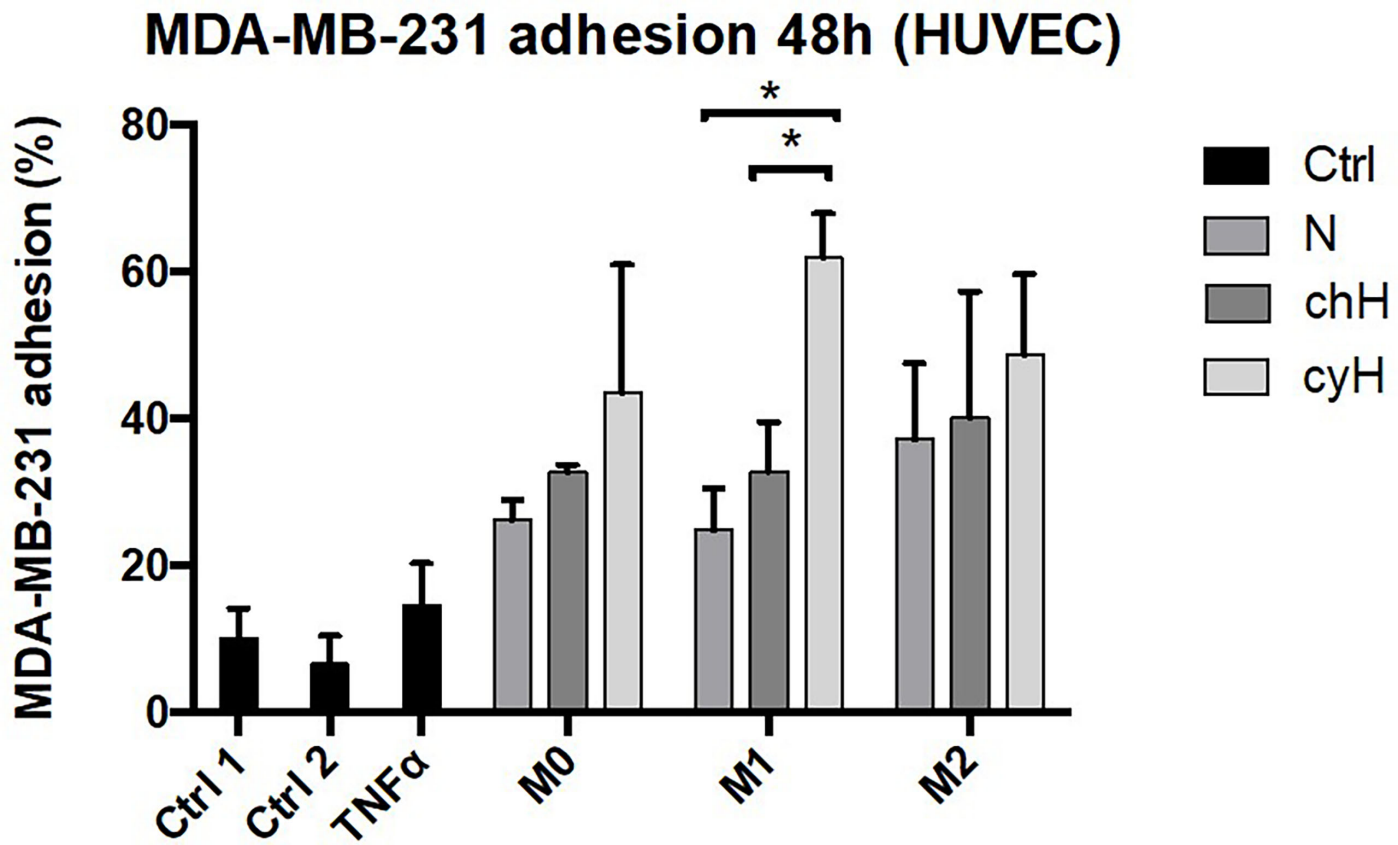
Adhesion of MDA-MB-231 cancer cells on HUVECs incubated with macrophage-conditioned media. THP-1–derived unpolarized, M1-like, and M2-like macrophages were exposed to normoxia (N), chronic hypoxia (chH), or cycling hypoxia (cyH) for 6 h and were then left for 16 h in normoxic air to produce macrophage-conditioned media. Confluent monolayers of HUVECs were incubated 48 h with macrophage-conditioned media. Thereafter, the adhesion of MDA-MB-231 cancer cells on HUVECs was assessed by adhesion assay (n = 3, mean ± 1 SEM). Ctrl 1 and Ctrl 2 correspond to endothelial cells incubated with CO_2_-independent medium or EGM-2 medium, respectively. Incubation of endothelial cells with TNFα (1 ng/ml) for 6 h was used as positive control. Left: Quantification of MDA-MB-231 adhesion of HUVECs. Right: Representative picture of adherent calcein-labeled MDA-MB-231 breast cancer cells on HUVECs incubated for 48 h with macrophage-conditioned media. Statistical analysis was performed using Student’s t test. *p < 0.05.

We showed that the impact of macrophages on EC adhesiveness for THP-1 monocytes was increased by cyH (**Figure 1**). Furthermore, we showed that endothelial ICAM1 expression and protein abundance were enhanced by cyH-exposed macrophage compared with N- or chH-exposed macrophages (**Figures 4, 5** and **Supplementary Figures 4, 5**). Some studies showed that macrophages increased the extravasation of cancer cells toward EC monolayer (36–38). Nonetheless, the effect of macrophages on EC adhesiveness for cancer cells is still unknown. Hence, we tested the impact of the incubation of HUVECs with unpolarized, M1-like, and M2-like macrophages exposed to N, chH, and cyH on breast cancer cell adhesion onto the endothelium (**Figure 6** and **Supplementary Figure 6**). Adhesion of MDA-MB-231 breast cancer cells onto HUVECs was higher in HUVECs incubated for 48 h with unpolarized, M1-like, and M2-like macrophages compared with HUVECs incubated with control medium (**Figure 6**). Furthermore, the adhesion of MDA-MB-231 cancer cells onto HUVECs was significantly higher in HUVECs incubated for 48 h with M1-like cyH medium compared with HUVECs incubated for 48 h with M1-like N and M1-like chH media (**Figure 6**).

M1 markers (CD80 and CD86) showed a better correlation with markers of endothelial activation compared with total macrophage marker (CD68) and with M2 markers (CD163 and CD206) in breast cancer samples. Indeed, the Pearson correlation coefficient of CD80 and CD86 with ICAM1 was 0.51 and 0.57, respectively, whereas the Pearson correlation coefficient of CD68, CD163, and CD206 (MRC1) with ICAM1 was 0.50, 0.47, and 0.39, respectively. Moreover, the Pearson correlation coefficient R-value of CD80 and CD86 with VCAM1 was 0.59 and 0.649, respectively, whereas the Pearson correlation coefficient of CD68, CD163, and CD206 was 0.56, 0.55, and 0.58, respectively (**Supplementary Table 2**).

Together, these results showed that the incubation of ECs with macrophage media increases their adhesiveness for breast cancer cells and that the effect of M1-like macrophages on EC adhesiveness for breast cancer cells is potentiated by cyH. Activation of ECs by M1-like macrophages also seems to occur in tumor samples.

## 4 Discussion

Tumor inflammation and metastasis are two hallmarks of cancers (13, 39) associated with bad prognosis and cyH, TAMs and ECs are critically involved in these processes (4, 8, 15–18). For example, EC adhesiveness for monocytes and cancer cells is likely involved in cancer metastasis. Indeed, cell adhesion of circulating carcinoma cells occurs predominantly in metastatic organs. Furthermore, several treatments, which *in vitro* diminished EC adhesion molecule expression, diminished also cancer cell adherence to EC, experimental metastasis, and spontaneous metastasis. Moreover, the recruitment of inflammatory monocytes increases the occurrence of breast-to-pulmonary and of bone metastasis. Previously, we showed that cyH induced tumor inflammation in a murine tumor model and induced macrophage and EC pro-inflammatory phenotype (8, 9). In this work, the impact of N-, chH-, and cyH-exposed macrophages on EC pro-inflammatory phenotype, EC adhesion molecule expression and EC ability to bind monocytes and cancer cells were investigated. We showed that cyH-exposed macrophages increased ICAM1 expression in ECs and that unpolarized cyH-exposed macrophages increased the expression and secretion of pro-inflammatory cytokines in ECs. ECs incubated with M1-like cyH medium were more prone to bind monocytes and cancer cells than those incubated with M1-like N and M1-like chH media. These two processes are involved in the regulation of tumor inflammation and cancer metastasis (16–18, 36, 40).

M0 macrophages exposed to cyH induced more strongly the expression and secretion of pro-inflammatory cytokines (IL-6 and IL-8) and protein (ESM1) by ECs, compared with unpolarized macrophages exposed to N and chH (**Figures 2**, **3**). Interestingly, IL-6 is strongly expressed by ECs in human and murine glioblastoma tumors (41, 42). Furthermore EC-derived IL-6 is a cytokine that favors macrophage M2-like polarization, at least in murine glioblastoma (41). M2-like macrophage polarization is associated with bad prognosis in most cancer types (43). Furthermore, ESM1 is an EC biomarker that is strongly expressed in tumor ECs in several murine tumor models (44). ESM1 expression in cancer is correlated with bad prognosis in human gastrointestinal and hepatocellular carcinomas (45). Furthermore, a study showed that ESM1 induces ICAM1 expression in ECs and that could explain the stronger induction of ICAM1 expression in EA.hy926 cells by cyH-exposed unpolarized macrophages than N- or chH-exposed unpolarized macrophages (46).

ICAM1 expression and protein abundance in ECs and VCAM1 and E-Selectin expression in ECs were mostly induced by M1-like macrophages and were least induced by M2-like macrophages (**Figures 4**, **5** and **Supplementary Figures 3–5**). This is consistent with a previous study showing that M1-like macrophages were more potent than M2-like macrophages to induce adhesion molecule expression in ECs (35). cyH enhanced the ability of each macrophage type to induce ICAM1 mRNA expression in HUVECs (**Figure 4B**). Furthermore, cyH enhanced the ability of M1-like macrophages to increase ICAM1 protein abundance in both EC types (**Figure 5** and **Supplementary Figure 4**), whereas unpolarized and M2-like macrophages exposed to cyH were more potent (than those exposed to N or chH) inducers of ICAM1 protein expression in EA.hy926 cells or in HUVECs, respectively (**Figures 5A, B**). Together, these results showed that cyH enhanced the ability of macrophages to induce endothelial ICAM1 protein abundance. Endothelial ICAM1 expression is involved in the metastatic cascade. Indeed, EC ICAM1 is involved in cancer cell adhesion and extravasation of several cancer cell types, which are two needed steps for the metastatic spread of cancer cells (47). Furthermore, ICAM1 expression is involved in melanoma cell infiltration into liver upon their injection into the tail vein of mice (48).

In this study, macrophages modulated EC phenotype toward one allowing monocyte and cancer cell binding to EC (**Figures 1**, **6** and **Supplementary Figures 2, 6**). This is consistent with a previous study showing that incubation of ECs with the conditioned media of inflammatory macrophages promoted monocyte adhesion onto endothelium (49). Interestingly, we showed in this work that the three macrophage types—namely, unpolarized, M1-like, and M2-like—were able to increase breast cancer cell binding onto endothelium (**Figure 6**). Furthermore, cyH enhanced the ability of macrophages (mostly M1-like macrophages) to induce monocyte and cancer cell binding onto endothelium (**Figures 1**, **6** and **Supplementary Figures 2, 6**). These two features are the first steps of their infiltration into tumors and are strongly involved in tumor metastasis (18, 36, 40). Indeed, monocyte (or macrophage) infiltration into secondary tumor sites strongly increases metastatic formation, notably *via* increasing cancer cell extravasation and seeding into secondary tumor site (36, 40). TAMs increase both cancer cell extravasation into secondary tumor sites and cancer cell intravasation at primary tumor site (at site called TME of metastasis) (19). On the other hand, cancer cell binding onto endothelium is a critical step of the metastatic cascade (18). The impact of macrophages on the ability of ECs to allow for the adhesion of cancer cells was poorly known. It was previously shown that pro-inflammatory macrophages promoted cancer cell binding onto lymphatic ECs (50), likely *via* IL-1β secretion, whereas macrophages treated with carcinoembryonic antigen (CEA) were able to promote colon carcinoma HT-29 cancer cell binding onto ECs (51), but no data were generated without pre-treatment of macrophages with CEA. Here, we showed for the first time that unpolarized, M1-like, and M2-like macrophages, on their own, were able to modulate EC phenotype toward one allowing breast cancer cell binding onto ECs and that the effect of M1-like macrophages was potentiated by cyH. It would be interesting to investigate the molecular mechanism involved in this process, such as molecules involved in EC-cancer cell interaction, and it is important to note that the increase in ICAM1 expression in ECs observed in this study could be involved in this process. Other EC proteins involved in cancer cell binding are notably N-cadherin, E-selectin, and integrins (18, 31). Monocytes and macrophages affect cancer cell extravasation notably *via* VEGF- and MMP9-mediated EC permeability (36, 37, 52). It would then be worth investigating the impact of cyH-exposed unpolarized, M1-like, and M2-like macrophages on cancer cell extravasation and EC permeability.

In this work, we show that the incubation of macrophages with cyH increased their ability to induce pro-inflammatory cytokine expression and secretion in ECs and ICAM1 expression in ECs and to shift EC phenotype toward one allowing the adhesion of monocytes and cancer cells. This effect is specific to cyH because it was not observed in ECs incubated with macrophages exposed to chH or N. Strikingly, cyH, on its own, induces similar effects to ECs than macrophages exposed to cyH (8, 46), except for cancer cell adhesion that was not investigated in ECs exposed to cyH to our knowledge. Short-term exposure of ECs to cyH (6 h) had an impact on EC alone with pro-inflammatory cytokine stimulation (8) because the effects were only observed with ECs incubated with TNFα, whereas the effects of the long-term exposure of ECs to cyH (> 48 h) were independent of pre-existing inflammation (46). Interestingly, to our knowledge, it is not known whether cyH, on its own, induces EC pro-inflammatory phenotype in the absence of pro-inflammatory cytokine stimulation, whereas cyH increases the ability of macrophages to induce pro-inflammatory phenotype in ECs in the absence of pre-treatment of ECs with pro-inflammatory cytokines. Because some common effects of cyH-exposed macrophages and cyH on ECs are shared, it would be interesting to investigate if some levels of synergy exist between the two kinds of stimulation. This synergy is very likely because macrophages (mostly unpolarized and M1 macrophages) secrete high levels of TNFα, and that TNFα is required to induce the effects of cyH on ECs during the short-term exposure to cyH (8). Furthermore, this is physiologically relevant because, in tumors, some macrophages—called perivascular macrophages—are localized near to ECs (53) and that these two cell types should hence be exposed together to cyH in the TME.

In conclusion, we compared the impact of unpolarized, M1-like, and M2-like macrophages on ECs. Furthermore, we studied the effects of the pre-exposure of macrophages with N, chH, and cyH on their impact on ECs. We showed that unpolarized, M1-like, and M2-like macrophages, on their own, shift EC phenotype toward one allowing cancer cell binding onto ECs and that this effect was enhanced in M1-like macrophages exposed to cyH (**Figure 6**). Together, these results further confirm that the previous results that we obtained prove that cyH induced tumor inflammation, promoted EC inflammation, and promoted a pro-inflammatory phenotype in human and murine unpolarized and M1-like macrophages (8, 9). These effects are specific to cyH because they are not observed with chH. In murine models and in human patients, tumor metastasis occurrence is increased by cyH (54, 55). Hence, cyH could promote cancer metastasis, at least in part, by increasing the ability of macrophages to trigger cancer cell and monocyte adhesion to ECs (and likely their extravasation) at secondary tumor sites. Furthermore, cyH could also promote the ability of macrophages to induce cancer cell intravasation at primary tumor site and, hence, metastasis. It would be interesting to investigate by which secreted molecules macrophages induce their effects on ECs and to investigate by which intracellular mechanisms macrophages induce these effects in ECs. Furthermore, it would be interesting to confirm that these effects are also observed in the presence of cancer cells or in the TME. This work provides interesting lines of investigation that could explain the promotion of metastasis and inflammation by cyH. This may lead to the discovery of new therapeutic strategies for the treatment of tumor inflammation and metastasis.

## Data availability statement

The raw data supporting the conclusions of this article will be made available by the authors, without undue reservation.

## Author contributions

VD performed the experiments and wrote the main manuscript. CH performed the bioinformatics analyses. OF designed the project and critically revised the manuscript. FS supervised the whole part performed on HUVECs and critically revised the manuscript. CM (corresponding author) designed the project, critically revised the manuscript, and supervised the entire work. All authors contributed to the article and approved the submitted version.

## Funding

This work was supported by TRANSUNIVInterreg,ERASMUS, and FNRS mobility grants.

## Acknowledgments

The authors are thankful to the technological platform Morph- Im (University of Namur). We thank Catherine Demazy for the pictures taken under confocal microscopy. Victor Delprat is a research fellow of Televie. Camille Huart is recipient of a Fonds National de la Recherche Scientifique (F.R.S.-FNRS, Belgium) PhD fellowship.The authors thank Dr.Anthony Treizebre forMDA-MB- 231 cells as a gift.

## Conflict of interest

The authors declare that the research was conducted in the absence of any commercial or financial relationships that could be construed as a potential conflict of interest.

## Publisher’s note

All claims expressed in this article are solely those of the authors and do not necessarily represent those of their affiliated organizations, or those of the publisher, the editors and the reviewers. Any product that may be evaluated in this article, or claim that may be made by its manufacturer, is not guaranteed or endorsed by the publisher.

## Abbreviations

chH, chronic hypoxia; cyH, cycling hypoxia; ECs, endothelial cells; ESM1, endothelial cell–specific molecule 1; HUVEC, human umbilical vein endothelial cells; ICAM1, intercellular adhesion molecule 1; MMP9, matrix metalloproteinase 9; N, normoxia; N-cadherin, neuronal cadherin; TAMs, tumor-associated macrophages; TME, tumor microenvironment; VCAM1, vascular cell adhesion molecule 1.
