# Supplementary material for: The impact of macrophages on endothelial cells is potentiated by cycling hypoxia: Enhanced tumor inflammation and metastasis
Source: Front Oncol. 2022 Sep 28;12:961753. doi: 10.3389/fonc.2022.961753 (PMC9554541; doi:10.3389/fonc.2022.961753)
Supplement: Supplementary file 1 [file DataSheet_1.pdf]

The impact of macrophages on endothelial cells is potentiated by cycling hypoxia: enhanced tumor inflammation and metastasis

Victor DELPRAT[1], Camille HUART[1], Olivier FERON[2], Fabrice SONCIN[3] and Carine MICHIELS[1*]

[1] Biochemistry and Cellular Biology Research Unit (URBC), Namur Research Institute for LIfe Sciences (NARILIS), University of Namur (UNamur), 61 Rue de Bruxelles, B-5000, Namur, Belgium.

Supplementary data

Primary antibodies		
Reference	Incubation time	Dilution
Rabbit polyclonal Ab anti-ICAM1 (H reactivity), CST, #4915	O/N 4°C	1/1 000
Mouse mAb anti-α-tubulin (H+M reactivity), Sigma, #T5168	30 min RT	1/10 000
**Secondary antibody**		
Reference	Incubation time	Dilution
Goat anti-mouse IgG IRDye conjugated, LI-COR, Biosciences, #926-69070 (680)	1h RT	1/10 000
Goat anti-rabbit IgG IRDye conjugated, LI-COR, Biosciences, #926-68071 (680)	1h RT	1/10 000

Supplementary Table S1. References of primary and secondary antibodies used for Western blot analyses

Gene A	Gene B	Pearson *r*	p-value
CD68	ICAM1	0.50355868	***
CD80	ICAM1	0.51002051	***
CD86	ICAM1	0.57074485	***
CD163	ICAM1	0.47114399	***
CD206	ICAM1	0.39275600	***
CD68	VCAM1	0.5629818	***
CD80	VCAM1	0.5927903	***
CD86	VCAM1	0.6497379	***
CD163	VCAM1	0.5550227	***
CD206	VCAM1	0.5833463	***

Supplementary Table S2. Correlation analysis of markers of macrophage (CD68, CD80, CD86, CD163 and CD206) with markers of endothelial activation (ICAM1 and VCAM1) in breast cancer cohort of TCGA. (**A**) Correlation Pearson *r* values were calculated for each combination from breast cancer cohort (***, $p < 0.001$).

A

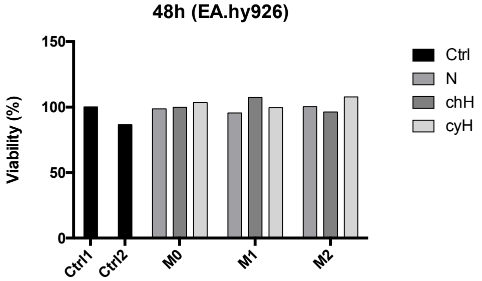

B

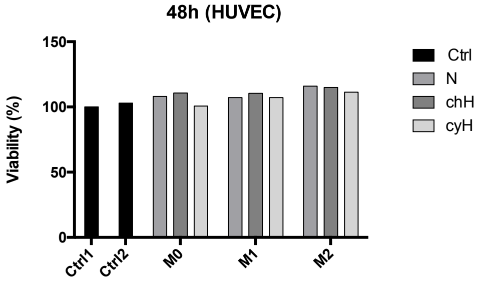

Supplementary Fig. 1. Endothelial cell viabiliity of EC exposed to macrophage-conditioned media during 48h. THP-1-derived M0, M1 and M2 macrophages were exposed to normoxia (N), chronic hypoxia (chH) or cycling hypoxia (cyH) during 6h and were then left for 16h in normoxic air in order to produce macrophage conditioned-media. Thereafter, EA.hy926 cells **(A)** and HUVEC **(B)** were incubated for 48h with macrophage conditioned-media and the viability of endothelial cells was assessed by MTT assay (n=1). Ctrl 1 corresponds to endothelial cells incubated with CO_2 independent medium. Ctrl 2 corresponds to endothelial cells incubated with DHGL-1 **(A)** or EGM-2 **(B)** medium.

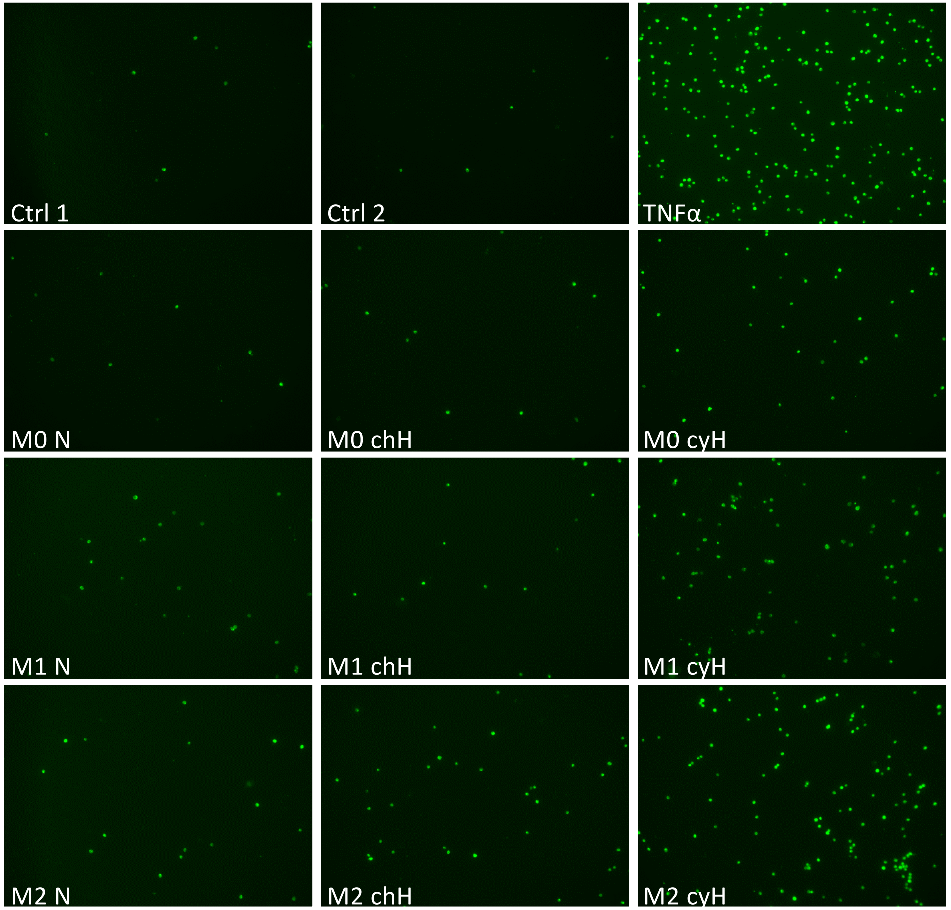

Supplementary Fig. 2. Pictures of adherent calcein-labeled THP-1 monocytes on HUVEC incubated 24h with macrophage conditioned-media (Fig. 1C)

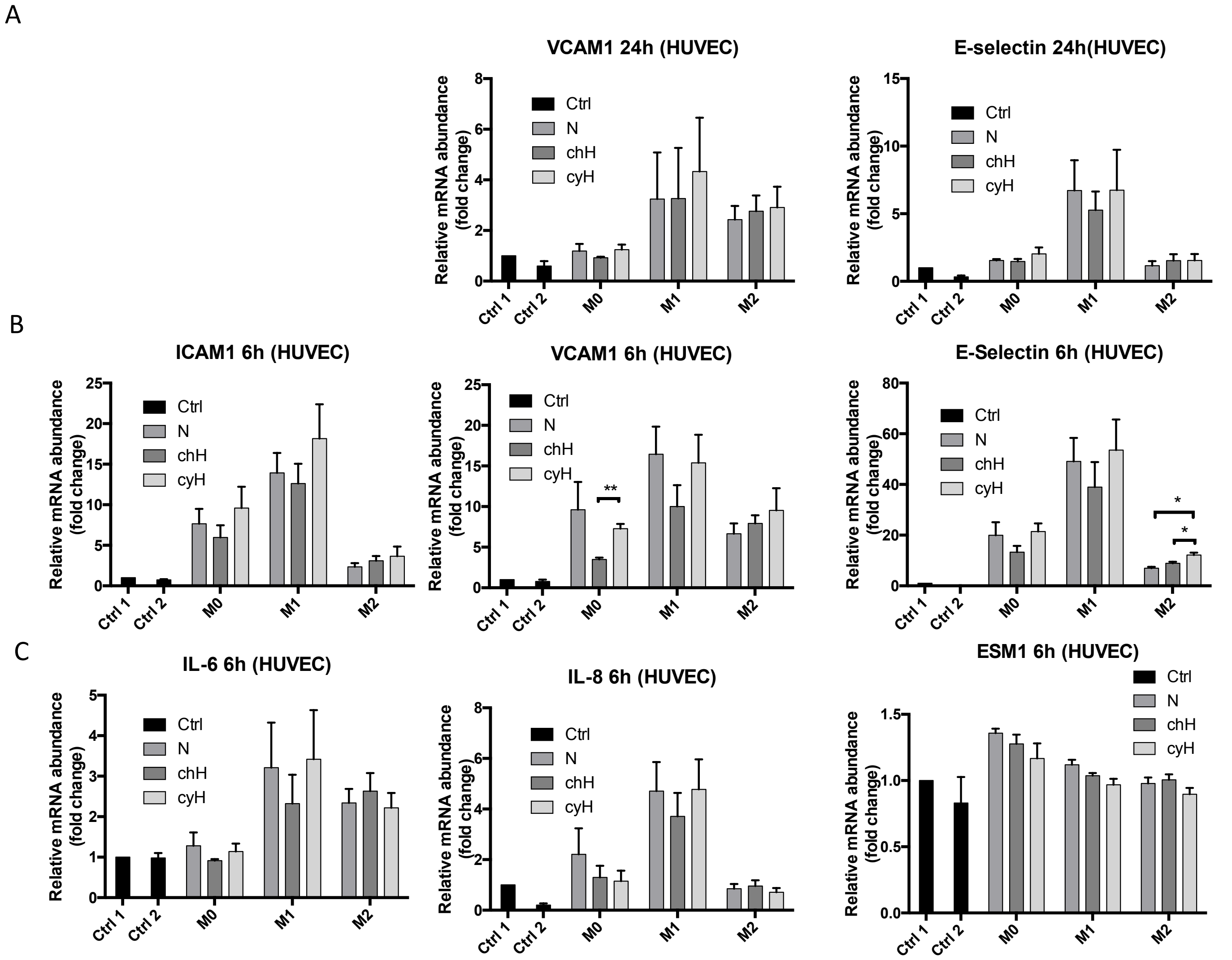

Supplementary Fig. 3. Endothelial adhesion molecule and pro-inflammatory mRNA expression in HUVEC incubated with macrophage conditioned-media. THP-1-derived M0, M1 and M2 macrophages were exposed to normoxia (N), chronic hypoxia (chH) or cycling hypoxia (cyH) during 6h and were then left for 16h in normoxic air in order to produce macrophage conditioned-media. Thereafter, HUVEC were incubated for 24h **(A)** or 6h **(B and C)** with macrophage conditioned-media. mRNA expression of endothelial adhesion molecules **(A and B)** and pro-inflammatory cytokines and gene expression **(C)** was assessed by RT-qPCR (n=4, mean ± 1 SEM). Ctrl 1 and Ctrl 2 correspond to endothelial cells incubated with CO_2 independent medium or EGM-2 medium, respectively. Statistical analysis was performed using Student's t test. *$p < 0.05$

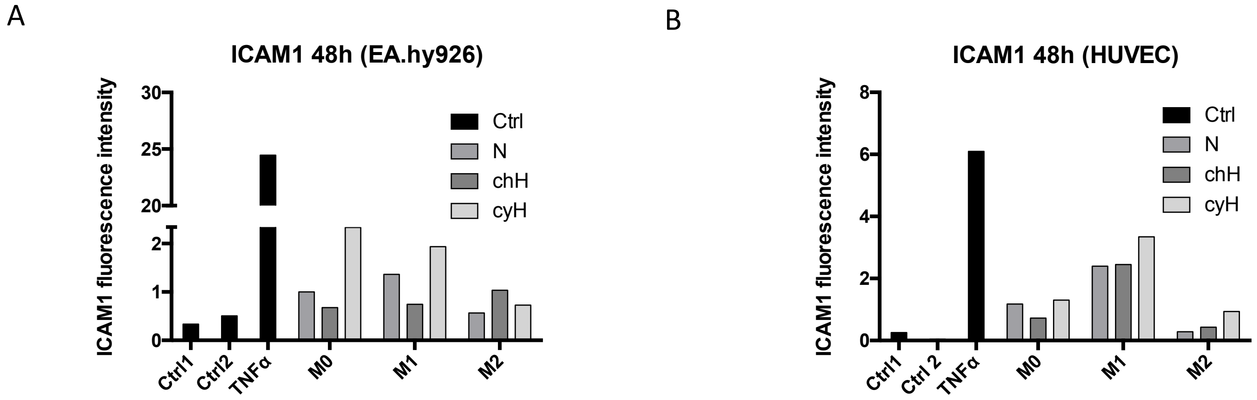

Supplementary Fig. 4. Quantification of ICAM1 immunofluorescence labeling intensity shown in figure 4. EA.hy926 (A) or HUVEC (B) were incubated 48h with macrophage conditioned-media, and ICAM1 immunofluorescence labeling was performed. These results are the mean of two independent experiments. n=2

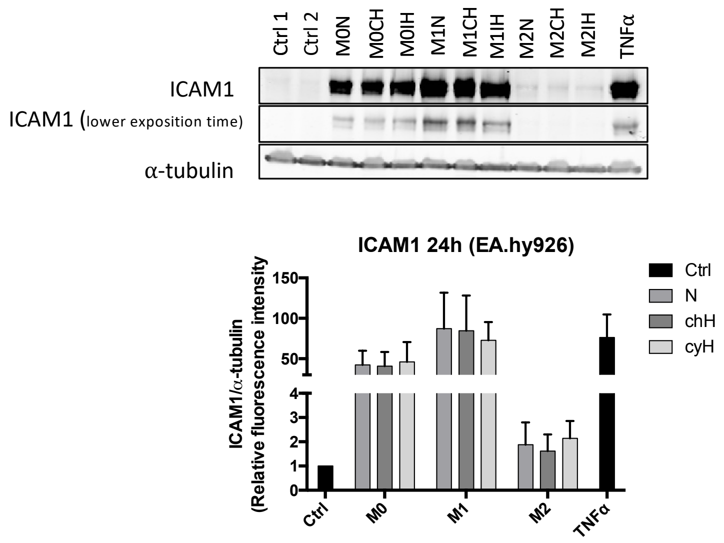

Supplementary Fig. 5. ICAM1 protein abundance in EA.hy926 incubated 24h with macrophage conditioned-media. THP-1-derived M0, M1 and M2 macrophages were exposed to normoxia (N), chronic hypoxia (chH) or cycling hypoxia (cyH) during 6h and were then left for 16h in normoxic air in order to produce macrophage conditioned-media. Thereafter, EA.hy926 cells were incubated for 24h with macrophage conditioned-media. ICAM1 protein abundance in EA.hy926 cells was analyzed by western blot (n = 3, mean ± 1 SEM). Ctrl corresponds to endothelial cells incubated with CO_2 independent medium. Incubation of endothelial cells with 1ng/mL TNFα during 16h was used as positive control. Statistical analysis was performed using Student's t test.

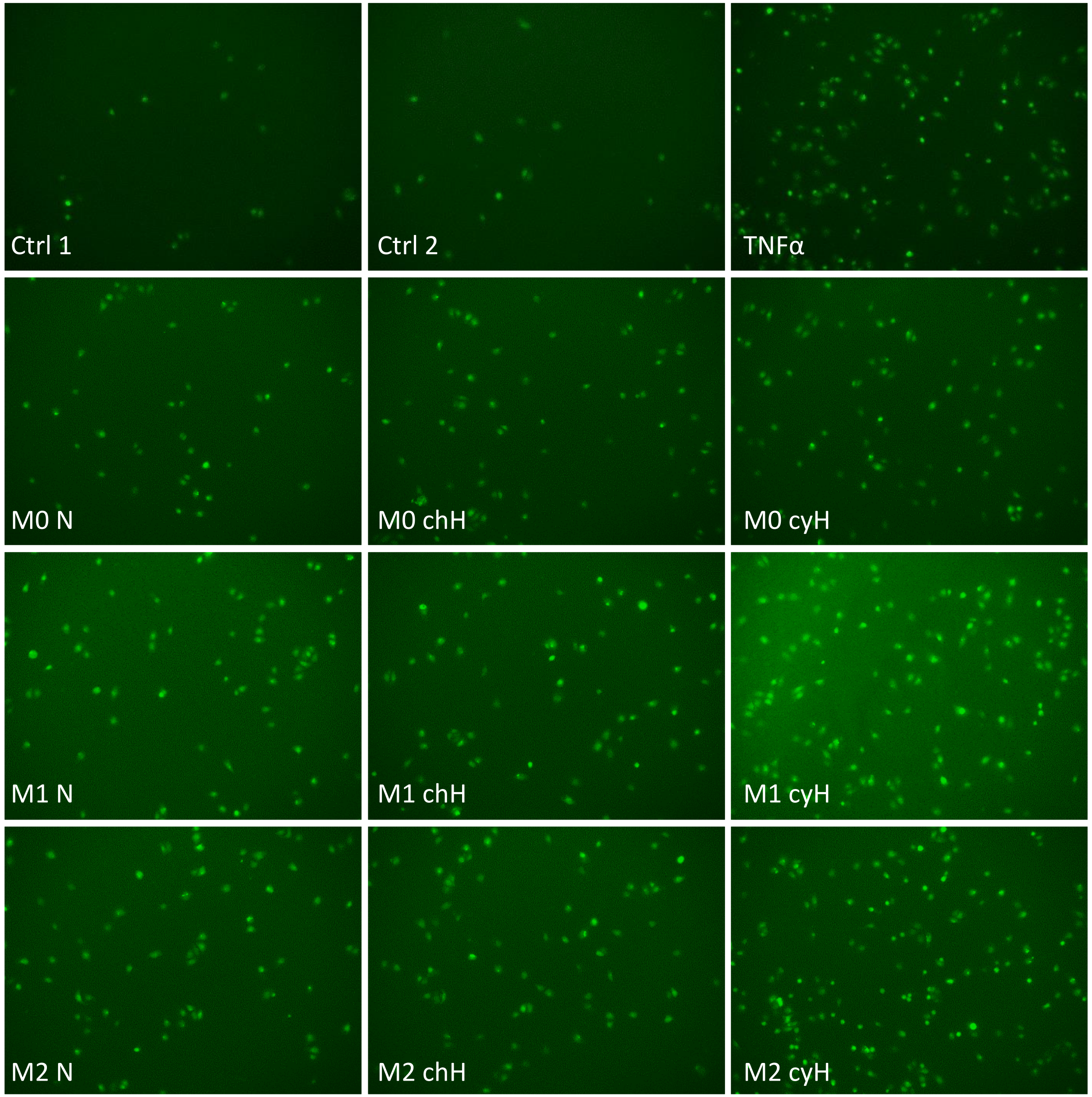

Supplementary Fig. 6. Pictures of adherent calcein-labeled MDA breast cancer cells on HUVEC incubated 48h with macrophage conditioned-media (Related to Fig. 6)
